# Caudal Approach to Laparoscopic Liver Resection—Conceptual Benefits for Repeated Multimodal Treatment for Hepatocellular Carcinoma and Extended Right Posterior Sectionectomy in the Left Lateral Position

**DOI:** 10.3389/fonc.2022.950283

**Published:** 2022-07-11

**Authors:** Tomoyoshi Endo, Zenichi Morise, Hidetoshi Katsuno, Kenji Kikuchi, Kazuhiro Matsuo, Yukio Asano, Akihiko Horiguchi

**Affiliations:** ^1^ Department of Surgery, Fujita Health University School of Medicine Okazaki Medical Center, Okazaki, Japan; ^2^ Department of Gastroenterological Surgery, Fujita Health University School of Medicine Bantane Hospital, Nagoya, Japan

**Keywords:** laparoscopic liver resection, caudal approach, postural change, repeat hepatectomy, hepatocellular carcinoma, chronic liver disease, posterior sectionectomy

## Abstract

We had reported the novel concept of “caudal approach in laparoscopic liver resection” in 2013. In the first report, the caudal approach of laparoscopic transection–first posterior sectionectomy without prior mobilization of the liver in the left lateral position was described. Thereafter, 10 complex laparoscopic extended posterior sectionectomies with combined resection of the right hepatic vein or diaphragm were performed using the same approach. In the present study, the short-term outcomes of these cases and 42 cases of laparoscopic sectionectomies or hemi-hepatectomies (excluding left lateral sectionectomy) were compared. There was no statistically significant difference between the groups in terms of patients’ backgrounds, diseases for resection, preoperative liver function, tumor number and size, as well as outcomes, operation time, intraoperative blood loss, morbidity, conversion to laparotomy, and post-operative hospital stay. Even complex laparoscopic extended posterior sectionectomy was safely performed using this procedure. This approach has the technical benefits of acquiring a well-opened transection plane between the resected liver fixed to the retroperitoneum and the residual liver sinking to the left with the force of gravity during parenchymal transection, and less bleeding from the right hepatic vein due to its higher position than the inferior vena cava. Furthermore, it has an oncological benefit similar to that of the anterior approach in open liver resection, even in posterior sectionectomy. The detailed procedure and general conceptual benefits of the caudal approach to laparoscopic liver resection for repeated multimodal treatment for hepatocellular carcinoma are described.

## Introduction

After the introduction of laparoscopic liver resection (LLR) in the early 1990s (1–3), the procedure had been rapidly developing with technical and instrumental improvements (4) through two international consensus conferences (5, 6) and three world congresses of the International Laparoscopic Liver Society (7). Partial resections in the anterolateral segments and left lateral sectionectomy have been established as common procedures. In addition, laparoscopic hemi-hepatectomies and sectionectomies (left-medial, right-anterior, and right posterior), which have straightforward caudal–cranial transection planes suitable for the laparoscopic approach, are the next-step candidates of LLR to get established as common procedures (6). Among them, anterior and medial sectionectomies have difficulty transecting a large area of the boundary plane on both the right and left sides. On the other hand, posterior sectionectomy has a specific difficulty in acquiring a good surgical field and bleeding control because the transection plane is horizontal and deep in the subphrenic space (rib cage) beneath the large and heavy right liver in the usual supine position.

**Figure 1:**
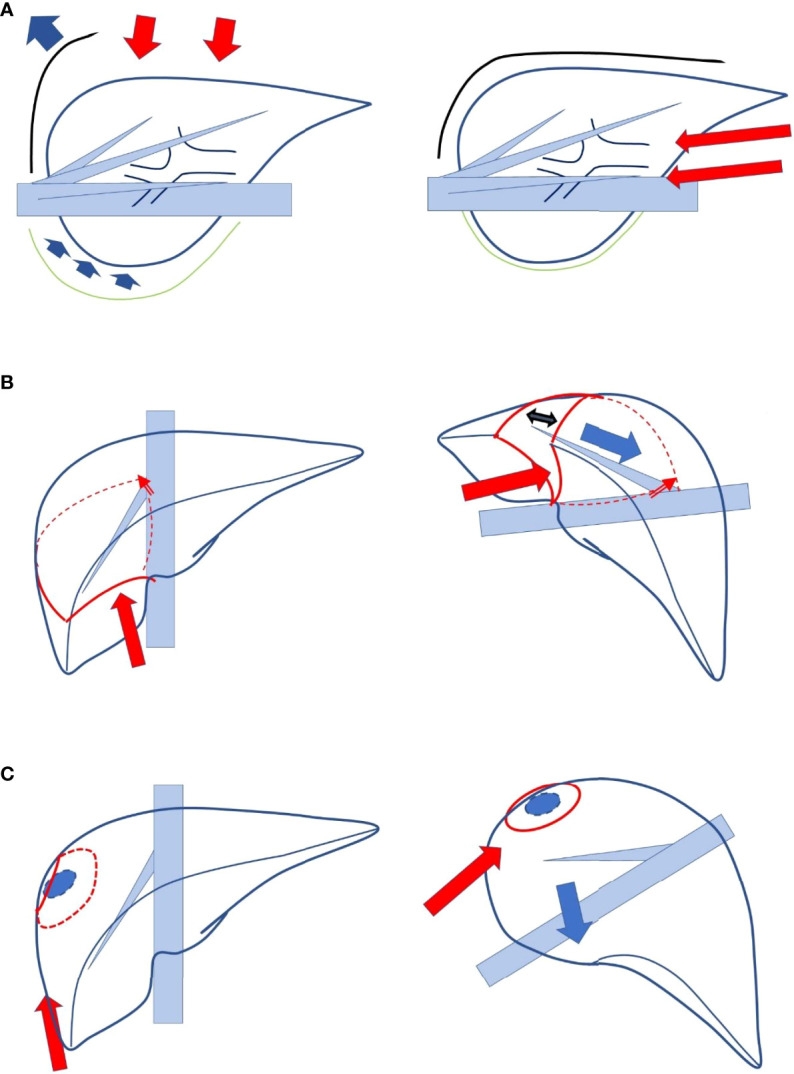
**(A)** Liver resection is a procedure in which the liver protected inside the subphrenic “rib cage” is handled and resected. In open liver resection, the cage is opened with the big subcostal incision followed by lifting the costal arch, and the mobilized liver is picked up from the retroperitoneum (left, lateral view). In a laparoscopic procedure, laparoscope and forceps intrude into the cage directly from caudal direction without destruction of the cage and with minimum mobilization of the liver (right, lateral view). **(B)** The boundary plane between the anterior and posterior sections, the cutting plane of posterior sectionectomy, is horizontal and the large heavy liver and gravity obstruct exposure of the plane in supine position (left). In the left lateral position with transection prior to mobilization, the cutting plane between the retroperitoneal-fixed resected liver and the sunk remnant liver is well-opened (right). **(C)** The transection of segmentectomy or partial resection in segment 7 of the liver should be performed in the deep small subphrenic space with segment 6 as an obstacle in the way to the lesions. In a semi-prone position, direct access to segment 7 can be obtained with the elimination of segment 6 in the downward left direction by gravity (left).

We had reported the novel concept of “caudal approach to LLR” in 2013 (8), which was followed by several researchers (9, 10), and it was defined as a main conceptual change from open liver resection (OLR) in the statement of the 2nd International Consensus Conference on LLR (6). In the first report, “caudal approach of laparoscopic transection–first posterior sectionectomy without prior mobilization of the liver in left lateral position” was described. Since the transection plane turns vertically and the plane is well opened between the retroperitoneal-fixed resected liver (posterior section) and the residual liver sunk down to the left with the force of gravity, a good surgical field is obtained in the procedure. In addition, upward standing of the right hepatic vein (RHV) from the inferior vena cava (IVC) on the transection surface decreases intravenous pressure, which leads to less bleeding. Using this approach, we performed even more complex procedures, such as laparoscopic extended posterior sectionectomy with combined resection of the RHV or diaphragm. Moreover, the caudal approach also has several conceptual benefits. Liver resection is a procedure of handling and resecting the liver protected inside the subphrenic “rib cage”. In OLR, the cage is opened with a large subcostal incision followed by costal arch lifting, and the mobilized liver is picked up from the retroperitoneum. In contrast, in the laparoscopic procedure, laparoscope and forceps intrude into the cage directly from the caudal direction without destruction of the cage and with minimal mobilization of the liver (with minimal damage to the adherent structures and the liver itself, **Figure 1A**).

In this perspective, we attempted to describe the current status of the caudal approach to LLR. The short-term outcomes of our laparoscopic extended posterior sectionectomy with combined resection of the RHV or diaphragm are compared to those of the other anatomical LLRs for sections or more (excluding left lateral sectionectomy), and the detailed procedure is described. In addition, the conceptual benefits of the caudal approach for repeated multimodal treatments of hepatocellular carcinoma (HCC) are discussed.

## Short-Term Outcomes of Laparoscopic Extended Posterior Sectionectomy With Combined Resection of Right Hepatic Vein or Diaphragm

After the first report on the caudal approach laparoscopic posterior sectionectomy, 10 complex laparoscopic extended posterior sectionectomies with combined resection of the RHV (nine cases) or diaphragm (one case) were performed using the same approach. Herein, the short-term outcomes of these 10 cases and the other 42 anatomical LLR cases for sections or more (excluding left lateral sectionectomy) were compared.

Background-related factors, including sex, age, and body mass index; the American Society of Anesthesiologists physical status classification of the patients; diseases for resection; preoperative liver functional indicators, including plasma levels of total bilirubin and albumin, platelet counts, prothrombin time, and indocyanine green retention rate at 15 min; tumor number and size; as well as postoperative short-term outcomes, including operation time, intraoperative blood loss, conversion to laparotomy, morbidity, and post-operative hospital stay were compared between the groups (**Table 1**).

**Table 1:** Comparison between laparoscopic extended posterior sectionectomy cases and laparoscopic liver resection cases for section or more in background factors and postoperative short-term outcomes.

	Extended Posterior Sectionectomy Cases, *n* = 10	Laparoscopic Liver Resection Cases for Section or More, *n* = 42	*p*-value
**BACKGROUND FACTORS**			
**Age (years)**	62.10 ± 12.20	68.52 ± 9.76	0.147
**Sex (Male: Female)**	6:4	32:10	0.300
**Diseases for resection** **(HCC:Mets:other)**	4:5:1	17:11:14	0.222
**Body mass index**	23.8 ± 2.1	22.8 ± 3.5	0.392
**ASA-PS (1:2:3)**	11:30:1	3:7:0	0.868
**ICG R15 (%)**	10.12 ± 4.89	9.91 ± 4.77	0.904
**Total bilirubin (mg/dl)**	0.60 ± 0.21	0.65 ± 0.27	0.506
**Albumin (g/dl)**	3.84 ± 0.48	3.88 ± 0.48	0.812
**Platelet (×10^4^/μl)**	21.45 ± 6.02	21.77 ± 9.36	0.893
**Prothrombin time (%)**	104.40 ± 12.76	102.56 ± 13.99	0.694
**Number of tumors**	1.90 ± 0.99	2.06 ± 2.20	0.750
**Size of tumor (mm)**	42.90 ± 19.18	57.87 ± 39.45	0.252
**SHORT-TERM OUTCOMES**			
**Operation time (min)**	499.00 ± 108.38	452.12 ± 127.12	0.253
**Intraoperative blood loss (ml)**	438.50 ± 425.50	746.43 ± 1523.415	0.261
**Conversion to laparotomy (no: yes)**	10:0	40:2	0.482
**Morbidity (no: yes)**	10:0	38:4	0.310
**Postoperative hospital stay (day)**	16.50 ± 6.13	23.24 ± 11.93	0.091

HCC, hepatocellular carcinoma; Mets, liver metastasis; ASA-PS, the American Society of Anesthesiologists physical status classification. Extended posterior sectionectomy, right posterior sectionectomy with the combined resection of the right hepatic vein (n = 9) or diaphragm (n = 1). Laparoscopic liver resection cases for section or more, and sectionectomy and hemi-hepatectomy cases excluding 10 extended posterior sectionectomy and left lateral sectionectomy. ICGR15, indocyanine green retention at 15 min. Morbidity, Clavien–Dindo grade 3 or above.

There was no statistically significant difference between the groups in terms of sex, age, body mass index, physical status class of the American Society of Anesthesiologists, diseases for resection, preoperative liver functional indicators (plasma levels of total bilirubin and albumin, platelet counts, prothrombin time, and indocyanine green retention rate at 15 min), and tumor number and size. In addition, for the short-term outcomes, there was no statistically significant difference between the groups in terms of operation time (499.00 ± 108.38 min vs. 452.12 ± 127.12 min in extended posterior sectionectomy vs. the other anatomical resections, *p* = 0.253), intraoperative blood loss (438.50 ± 425.50 ml vs. 746.43 ± 1,523.415 ml, *p* = 0.261), conversion to laparotomy (0/10 vs. 2/40 cases, *p* = 0.482), and post-operative hospital stay (16.50 ± 6.13 days vs. 23.24 ± 11.93 days, *p* = 0.091). Four cases in the anatomical LLR for section or more and zero case in the extended posterior sectionectomy groups developed major complications of grade III or above in the Clavien–Dindo classification; however, the difference was not significant (*p* = 0.310).

There are few reports on perioperative outcomes of laparoscopic major hepatectomy (hemi-hepatectomies and sectionectomies, excluding left lateral sectionectomy, and the same patient group as our anatomical LLR group). Takahara et al. analyzed the data of 929 patients in the Japanese registry and reported an intraoperative blood loss of 865.4 ml, an operation time of 441.3 min, and a complication rate of 16.4% (11). Most recently, in a study conducted by Chin et al. on 130 patients in a single high-volume center, an intraoperative blood loss of 500 ml, an operation time of 362.5 min, and a complication rate of 26.9% were reported (12). Our outcomes of anatomical LLR are comparable to those previously reported, and there are no significant differences between our outcomes in the extended posterior section LLR group and the anatomical LLR group. Although extended posterior sectionectomy with combined resection of the RHV or diaphragm is a complex procedure, the procedure is feasible and was performed safely using the caudal approach with short-term outcomes comparable to those of other anatomical LLRs for sections or more.

## Detailed Procedure and Benefits of the Caudal Approach to Laparoscopic Posterior Sectionectomy

Our LLR for posterior sectionectomy, including the extended one, is performed by placing patients in the left lateral or semi-lateral position with rotation to the left. Furthermore, liver parenchymal transection prior to mobilization is employed to obtain a well-opened transection plane.

In the first step of the procedure, dissection of the falciform and coronal ligaments is performed to increase the movability of the residual liver, which causes the liver to sink due to the force of gravity in the left direction, and results in a well-opened transection plane in the left lateral position. The root surfaces of the right and middle hepatic veins and the fissure between them are continuously exposed. The fissure is the endpoint of liver parenchymal transection in extended posterior sectionectomy combined with RHV resection. Thereafter, the hepatoduodenal ligament is encircled with vessel tape for the extracorporeal Pringle’s maneuver.

Without mobilization of the liver from the retroperitoneum, the liver parenchyma (between segments 6 and 1) above the IVC is transected to expose the dorsal surface of the posterior Glissonian pedicle and IVC. In addition, the anterior surface of the Glissonian pedicle is dissected from the liver parenchyma at Rouviere’s sulcus. Thereafter, the posterior Glissonian pedicle (or at least the Glissonian pedicle to segment 6) can be clamped with a bulldog clamp without the necessity of encircling and taping it.

According to the ischemic demarcation line, the liver parenchymal transection starts from the caudal edge of the liver. The demarcation line, IVC behind the liver, and root of the RHV are guides of the transection direction.

After the transection line reaches the level of Rouviere’s sulcus, the posterior Glissonian pedicle is encircled and divided. The peripheral parts of the RHV are then searched and exposed on the well-opened transection plane. Exposure of the peripheral parts of the RHV eventually leads to the exposure of the main branch surface. The RHV main branch is one of the guides of the transection direction accompanied by IVC, demarcation line, and the root of the RHV as the endpoint.

The transection following the RHV and IVC eventually reaches the confluence of the veins and diaphragm, and the liver parenchymal transection is completed. After completion of liver parenchymal transection, the RHV on the transection surface of the resected liver is divided for extended posterior sectionectomy with combined resection of the RHV. Finally, retroperitoneal dissection of the liver is performed and the right posterior section, with or without the RHV, is removed.

The following are the technical benefits of this approach:

1. A well-opened transection plane between the resected posterior section fixed to the retroperitoneum and the residual liver sinking to the left with the force of gravity during parenchymal transection can be acquired.

2. Less bleeding from the RHV due to its lower intravenous pressure caused by its higher position than the IVC can be accomplished. Furthermore, the blood from the vertical transection surface runs down, and a clear view of the bleeding point can be obtained.

3. The exposed RHV on the transection surface and IVC at the bottom of the surgical field, accompanied by the liver surface ischemic line after clamping the posterior Glissonian pedicle, can be a good guide for transection direction.

Furthermore, this procedure, as a non-touch isolation procedure similar to the anterior approach in OLR, has an oncological benefit not only in hemi hepatectomy like in OLR, but also even in posterior sectionectomy.

## Perspectives of Caudal Approach to LLR

### Caudal Approach and Postural Changes in Various LLRs

Currently, all LLR procedures are performed using the caudal approach with postural changes in our institution. Caudal approach in the head-up supine to left semi-lateral position have usually been employed for hemi-hepatectomies, anterior/medial sectionectomies, and LLRs of anterolateral segments, segment 8, cranial 4 and 1. The left lateral position is applied for posterior sectionectomy, and the semi-prone position is applied for segment 7.

As mentioned above, the boundary plane between the anterior and posterior sections, that is, the cutting plane of posterior sectionectomy, is horizontal in the supine position. Although the cutting plane should be well opened in the small subphrenic “rib cage” for a safe and stable LLR, the large and heavy right liver and gravity obstruct the exposure of the cutting plane of posterior sectionectomy in this position. In contrast, a clear view from the caudal direction (**Figure 1A**) is among the advantages of LLR. Therefore, we developed a procedure that facilitates the exposure of the cutting plane: a caudal approach laparoscopic posterior sectionectomy with parenchymal transection prior to mobilization in the left lateral position. In this procedure, the cutting plane between the retroperitoneal-fixed resected liver and the sunk remnant liver is well opened (**Figure 1B**). Moreover, transection in segmentectomy or partial resection of segment 7 should be performed in the deeper and smaller subphrenic space, with segment 6 as an obstacle in the way to lesions under the laparoscopic caudal view even in the left lateral position. Therefore, we employed the semi-prone position for the LLRs, in which direct access to segment 7 can be obtained by eliminating segment 6 in the downward and left direction due to gravity (13, 14) (**Figure 1C**). For a large tumor lodged into the diaphragm, parenchymal transection prior to mobilization is performed to acquire a good view and manipulation on the transection plane well opened by the force of gravity, similar to posterior sectionectomy in the left lateral position. Regular segmentectomy and partial resection of segment 7 are performed after the mobilization of liver from the retroperitoneum. Mobilization leads to an adequate surgical space above the liver, and stable handling of tumors and instruments is established in the area of the RHV root. Although the area is at the bottom of the abdominal cavity in the supine position, it turns to be at the top of the abdominal cavity in the semi-prone position. The movements of the instruments are relatively restricted in our semi-prone caudal approach, without intercostal ports. However, using the port, in the paravertebral area to Morrison’s fossa, makes the manipulation feasible and stable with minimal risk of postoperative pleural effusion (14).

### Conceptual Benefits for Repeated Multimodal Treatments in Hepatocellular Carcinoma Patients With Chronic Liver Disease

Since HCC patients mostly have underlying chronic liver diseases (CLDs), they have a higher risk of post-treatment short-term morbidity and, in the long term, have a potential need for repeat treatments for metachronous multicentric lesions from the preneoplastic background liver, and risk of liver insufficiency during the long-repeated treatment course. When considering liver resection for HCC patients with CLD, not only oncological therapeutic effects on the current tumor but also residual liver function and the degree of invasive surgical stress, especially in the diseased liver, should be considered (15, 16). Patients who undergo liver resection are exposed to three different types of surgical stress (1): general, whole-body usual surgical stress (2); decreased liver function due to reduced liver volume after resection; and (3) surgery-induced injury to structures surrounding the liver and residual liver parenchyma (such as destruction of collateral blood and lymphatic flows by laparotomy, mobilization, and parenchymal injury caused by compression during liver resection) (17). Reduction of the third stress by the laparoscopic-specific caudal approach (8–10) in LLR (**Figure 1A**), especially for patients with HCC and CLD, decreases short-term morbidity (17, 18) and may also decrease deterioration of liver function after surgery, resulting in a decreased number of deceased patients with liver insufficiency and better accessibility to repeat multimodal treatments of metachronous lesions (19).

The impact of LLR on this issue depends on the background CLD severity, operative procedures (such as extent of dissection of the peritoneal attachments and adhesions), and resection volume of the functioning liver. Our previous study evaluated the short-term outcomes of liver surface small LLR in patients with severe CLD (Child Pugh, B/C; and indocyanine green retention rate at 15 min, ≥ 40%) (20). It revealed comparable short-term outcomes in patients with severe CLD compared to those with mild-to-moderate CLD. These surgeries were performed with direct access to the surface tumors and minimum dissection of attachments and adhesions, even without inflow control, and without touching any associated structures around the tumor. Only a laparoscopic approach, not an open approach, can make this resection setting possible. Patients with small surface tumors outside the bare area, without the need for dissection of peritoneal attachments on the surface and major vessels at the bottom, could benefit from LLR. However, the survival benefits of these treatments have not been proven (21). We, with Ghent University in Belgium as a primary investigating center, also performed the international retrospective study using propensity score matching analysis of patients with Child-Pugh B cirrhosis who underwent liver resection. The study showed that LLR is beneficial for patients with Child-Pugh B cirrhosis compared to open procedures (22, 23). Furthermore, LLR is speculated to have a benefit of less deterioration of liver function after surgery due to smaller damage related to surgical manipulation mentioned before (19, 24), which can lead to long-term benefits during repeated treatment history in HCC/CLD patients.

The treatment of repeat lesions, thereafter, is another major issue for the treatment strategy of HCC/CLD patients, as they harbor the potential for multicentric metachronous lesions occurring from the preneoplastic background. Modifications of the anatomy and the formation of adhesions increase the difficulty of repeat liver resection. The laparoscopic approach makes subsequent surgeries easier because of less adhesion formation (25). Furthermore, LLR allows for better visibility and manipulation in a small operative field between adhesions under the condition of repeat resection (26), which makes total adhesiolysis unnecessary in repeat LLR. We conducted an international retrospective collective study with propensity score matching analysis for repeat liver resection, comparing laparoscopic and open procedures (27, 28). It has been shown that laparoscopic repeat liver resection is feasible and has the short-term advantages of less intraoperative blood loss and less morbidity for selected patients. The overall survival curves after laparoscopic and open repeat liver resections were clearly separated with a better tendency in the laparoscopic group, although the disease-free survival curves were identical. The overall survival of HCC patients with CLD after liver resection is determined not only by the recurrence of the resected HCC but also by metachronous multicentric HCCs and liver insufficiency during postoperative long-term repeat treatment course (29, 30). During the long repeated treatment history of HCC/CLD patients, they should have sufficient residual liver function after each treatment, making it possible to undergo repeat treatments. We hypothesized that better overall survival after laparoscopic repeat liver resection may be caused by less deterioration of liver function (27), which made the repeat multimodal treatments more accessible, accompanied by less adhesion, and the number of deceased patients due to liver insufficiency decreased. The laparoscopic view and manipulation in the caudal approach (**Figure 1A**) facilitates better access in a small operative field between adhesions and reduces the need for adhesiolysis. This could be explained similarly to the advantage of LLR for patients with CLD noted previously. LLR, using its specific caudal approach, has conceptual benefits for HCC/CLD patients as a unique strong local treatment that makes repeated multimodal treatments more accessible. However, repeated LLRs have specific disadvantages. Disorientation can easily occur owing to the lack of tactile sensation and the lack of an overview of the entire operative abdominal field. Simulation and navigation from pre- and intra-operative imaging studies and well-planned small anatomical resection to secure tumor localization in the resected area and the tumor-free resected margin are used to overcome this disadvantage (24).

## Conclusion

The caudal approach, which is the basic approach to LLR, can be applied to a variety of LLRs using postural changes, even in complex procedures such as extended posterior sectionectomy with combined resection of the RHV or diaphragm. Its conceptual benefits could make repeated multimodal treatments more accessible and result in longer survival in patients with HCC/CLD.

## Data Availability Statement

The raw data supporting the conclusions of this article will be made available by the authors, without undue reservation.

## Ethics Statement

The studies involving human participants were reviewed and approved by Fujita health university ethics committee. Written informed consent for participation was not required for this study in accordance with the national legislation and the institutional requirements.

## Author Contributions

TE wrote the draft. ZM supervised the whole process from data collection to writing the manuscript and revised the final draft. HK, KK, KM, YA, and AH collected the data, discussed the data during data analysis, and wrote the draft. All authors contributed to the article and approved the submitted version.

## Conflict of Interest

The authors declare that the research was conducted in the absence of any commercial or financial relationships that could be construed as a potential conflict of interest.

## Publisher’s Note

All claims expressed in this article are solely those of the authors and do not necessarily represent those of their affiliated organizations, or those of the publisher, the editors and the reviewers. Any product that may be evaluated in this article, or claim that may be made by its manufacturer, is not guaranteed or endorsed by the publisher.
